# Comments on: Central serous chorioretinopathy after dacryocystorhinostomy operation on the same side

**DOI:** 10.4103/0301-4738.60071

**Published:** 2010

**Authors:** Parthasarathi Roy

**Affiliations:** Department of Ophthalmology, Haldia Sub-divisional Government Hospital, Purba Medinipur, West Bengal, India

Dear Editor,

I read with interest the article entitled “Central serous chorioretinopathy after dacryocystorhinostomy operation on the same side” by Mondal *et al*.[1] However, I have a strong reservation to accept that the central serous chorioretinopathy (CSCR) in the said case was due to the action of xylometazoline nasal drop or stress related to dacryocystorhinostomy (DCR) operation. Let me explain in the following few lines:

Each and every external DCR operation performed on earth is associated with some amount of operative stress on the patient, and also associated with the routine preoperative and postoperative use of nasal decongestant drops like xylometazoline/oxymetazoline. There was nothing in the article to suggest extraordinary stress like prolonged operative time, excessive bleeding or patient factors like highly stung (Type A) personality, nor there was anything to suggest prolonged use or excessive systemic absorption of the drug. In contrast, the drug was used for only 1 day (first postoperative day) before the symptoms of CSCR appeared on the second postoperative day.

Therefore the said case was one of the few millions of routine external DCR operations performed throughout the world for many years but without a single reported incidence of CSCR. A complication as startling as CSCR would have been reported, had it ever occurred in any part of this world (PubMed search, “Dacryocystorhynostomy and Central serous chorioretinopathy”–no results).

According to Karch *et al*.[2] as referred to in the *Laurence Book of Clinical Pharmacology* (7^th^ edition), the following criteria have to be fulfilled before attributing a cause-effect relationship between a drug and an adverse event:

1. Time sequence from taking drug is reasonable.
2. Event corresponds to what is known of drug.
3. Event ceases on stopping drug.
4. Event returns on restarting drug (rarely advisable).

However, none of the criteria have been fulfilled in the reported case:

1. It does not seem reasonable to attribute a cause-effect relationship following only 1 day of use of the xylometazoline nasal drop in an adult with a mature system to deal with a drug. The authors also did not mention why the xylometazoline nasal drop was started on the first postoperative day when it is customary to start it 7 days before operation and continue for another 7 days after operation.
2. CSCR was not known to occur after millions of routine DCR operations that took place in this world where similar stress and drugs were operant.
3. Resolving of the CSCR after 1 month cannot be attributed to the cessation of the drug as CSCR can naturally resolve after 1 month.[3]

Therefore, in my opinion the reported CSCR was mere a chance association rather than the side-effects of stress and xylometazoline nasal drop.
